# Tris[2,4-dichloro-6-(ethyl­imino­meth­yl)phenolato-κ^2^ *N*,*O*]cobalt(III)

**DOI:** 10.1107/S160053681203067X

**Published:** 2012-07-10

**Authors:** Qiu Ping Huang, Jing Jing Guo, Yi Dong Zhang, Shu Hua Zhang

**Affiliations:** a College of Chemistry and Bioengineering, Guilin University of Technology, Key Laboratory of Non-ferrous Metal Materials and Processing Technology, Ministry of Education, Guilin, 541004, People’s Republic of China

## Abstract

The asymmetric unit of the title compound, [Co(C_9_H_8_Cl_2_NO)_3_], contains three independent mol­ecules. In each mol­ecule, the Co^III^ ion is coordinated by an O atom and an N atom from three bidentate 2,4-dichloro-6-(ethyl­imino­meth­yl)phenolate ligands in a slightly distorted octa­hedral environment. In the crystal, a weak C—H⋯Cl hydrogen bond is observed.

## Related literature

For the crystal structures of related Co^III^ complexes, see: Park *et al.* (2008 ▶); Huang *et al.* (2010 ▶). For background to Schiff base compounds, see: Gupta & Sutar (2008 ▶); Sreenivasulu *et al.* (2005 ▶); Zhang & Feng (2010 ▶); Chen *et al.* (2011 ▶).
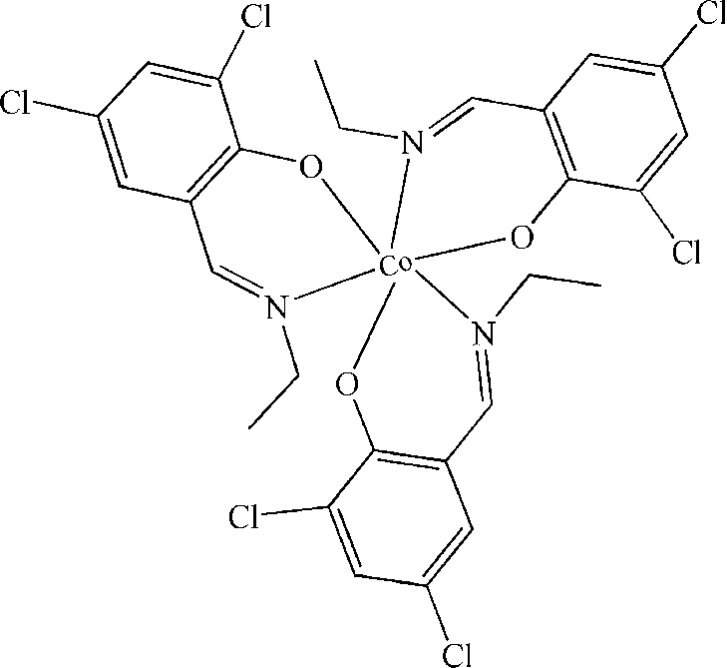


## Experimental

### 

#### Crystal data

[Co(C_9_H_8_Cl_2_NO)_3_]
*M* *_r_* = 710.12
Monoclinic, 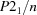

*a* = 13.7293 (12) Å
*b* = 45.892 (4) Å
*c* = 14.8108 (13) Å
β = 92.828 (1)°
*V* = 9320.4 (14) Å^3^
*Z* = 12
Mo *K*α radiation
μ = 1.10 mm^−1^
*T* = 296 K
0.38 × 0.16 × 0.12 mm

#### Data collection

Bruker SMART CCD diffractometer
Absorption correction: multi-scan (*SADABS*; Bruker, 2004 ▶) *T*_min_ = 0.806, *T*_max_ = 0.879
57477 measured reflections
21227 independent reflections
12089 reflections with *I* > 2σ(*I*)
*R*_int_ = 0.036

#### Refinement

*R*[*F* ^2^ > 2σ(*F* ^2^)] = 0.061
*wR*(*F* ^2^) = 0.171
*S* = 1.01
21227 reflections
1090 parameters
H-atom parameters constrained
Δρ_max_ = 0.56 e Å^−3^
Δρ_min_ = −0.46 e Å^−3^

### 

Data collection: *SMART* (Bruker, 2004 ▶); cell refinement: *SAINT* (Bruker, 2004 ▶); data reduction: *SAINT*; program(s) used to solve structure: *SHELXS97* (Sheldrick, 2008 ▶); program(s) used to refine structure: *SHELXL97* (Sheldrick, 2008 ▶); molecular graphics: *SHELXTL* (Sheldrick, 2008 ▶); software used to prepare material for publication: *SHELXTL*.

## Supplementary Material

Crystal structure: contains datablock(s) I, global. DOI: 10.1107/S160053681203067X/lh5498sup1.cif

Structure factors: contains datablock(s) I. DOI: 10.1107/S160053681203067X/lh5498Isup2.hkl

Supplementary material file. DOI: 10.1107/S160053681203067X/lh5498Isup3.cdx

Additional supplementary materials:  crystallographic information; 3D view; checkCIF report

## Figures and Tables

**Table 1:** Hydrogen-bond geometry (Å, °)

*D*—H⋯*A*	*D*—H	H⋯*A*	*D*⋯*A*	*D*—H⋯*A*
C44—H44*A*⋯Cl10^i^	0.97	2.80	3.748 (6)	168

Symmetry code: (i) 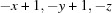.

**Fig. 1.**
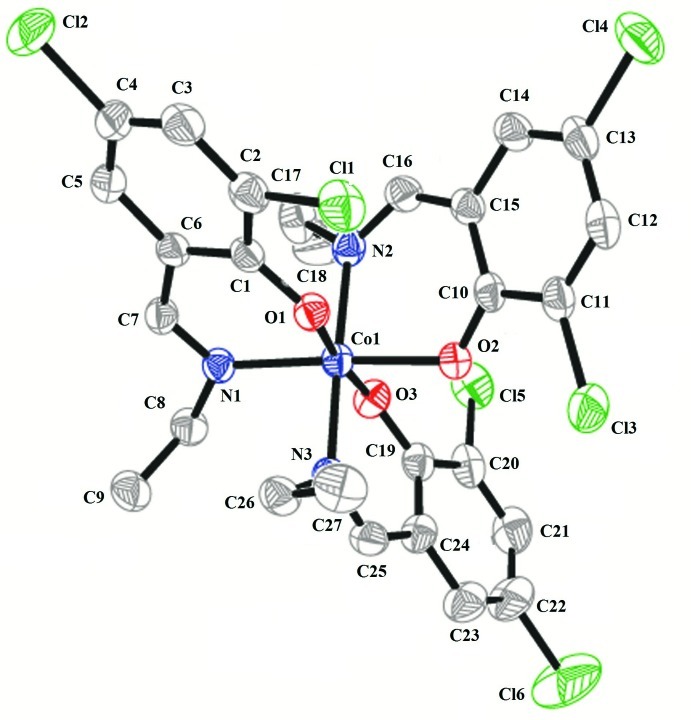
The molecular structure of the first independent molecule with ellipsoids drawn at the 30% level.

**Fig. 2.**
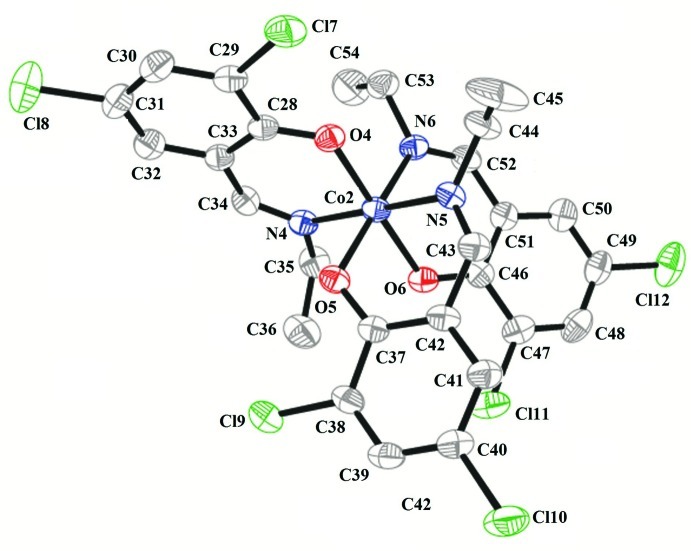
The molecular structure of the second independent molecule with ellipsoids drawn at the 30% level.

**Fig. 3.**
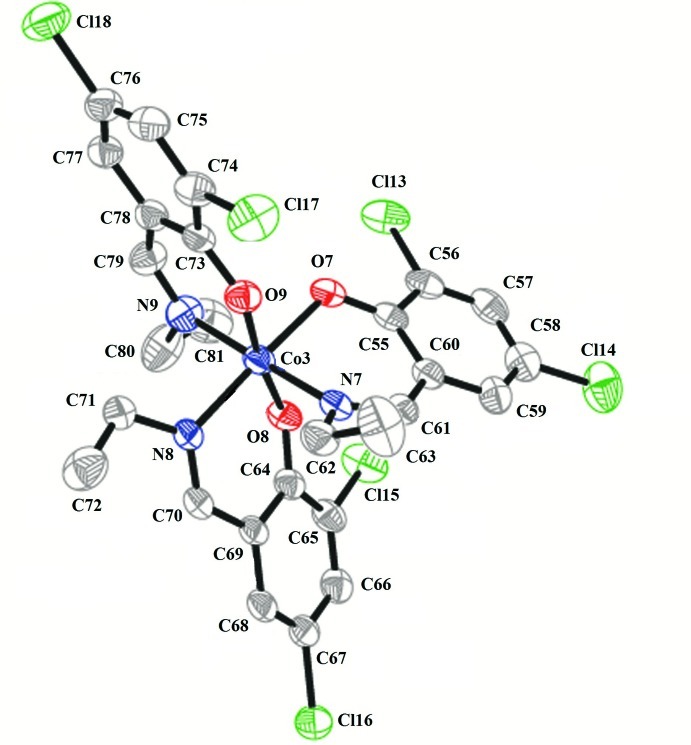
The molecular structure of the third independent molecule with ellipsoids drawn at the 30% level.

## supplementary crystallographic information

### Comment

Schiff base compounds have been studied for many years (Gupta & Sutar, 2008; Sreenivasulu *et al.* 2005; Zhang & Feng, 2010; Chen, *et al.* 2011) and have attracted interest because of their anticancer, catalytic and fluorescent properties. Using 3,5-dichloro-2-hydroxy-benzaldehyde, ethylamine and Co(ClO_4_)_2_.6H_2_O, we have hydrothermally prepared the title compound (I). Related Co^III^ complexes have already been structurally characterized (Park *et al.* 2008; Huang, *et al.* 2010).

In the title compound there are three independent molecules in the asymmetric unit. In each, the Co^III^ ion is coordinated by three O atoms and three N atoms from three bidentate *L* ligands forming a slightly distorted octahedral geometry (see Figs. 1, 2 and 3). The Co ion is in the 3+ oxidation state, as evidenced by bond valence summation calculations, charge balance considerations, and the presence of typical bond lengths for Co^III^ ions (Park, *et al.* 2008; Huang, *et al.* 2010). In the crystal, a weak C–H···Cl hydrogen bond is observed.

### Experimental

The title compound was prepared from a mixture of 3,5-Dichloro-2-hydroxy-benzaldehyde (0.185 g, 1 mmol), ethylamine solution (0.5 ml), Co(ClO_4_)_2_.6H_2_O (0.360g, 1 mmol), and methanol (8 ml) sealed in a 15 ml Teflon-lined stainless steel bomb, and kept at 393 K for 72 h under autogenous pressure. After the reaction was slowly cooled to room temperature, red needles were produced (yield: 56%, based on 3,5-Dichloro-2-hydroxybenzaldehyde). Anal. Calcd for C_81_H_72_Co_3_Cl_18_N_9_O_9(_%):*C* 46.66, H, 3.41; N, 5.91. Found (%): C, 46.61; H, 3.47; N,5.94.

### Refinement

H atoms were positioned geometrically and refined in a riding-model approximation, with distances C—H = 0.93 - 0.97Å and *U*_iso_(H) = 1.2 *U*_eq_(C) or *U*_iso_(H) = 1.5 *U*_eq_(C_methyl_).

### Crystal data

**Table:**

[Co(C_9_H_8_Cl_2_NO)_3_]	*F*(000) = 4320
*M**_r_* = 710.12	*D*_x_ = 1.518 Mg m^−^^3^
Monoclinic, *P*2_1_/*n*	Mo *K*α radiation, λ = 0.71073 Å
Hall symbol: -P 2yn	Cell parameters from 21227 reflections
*a* = 13.7293 (12) Å	θ = 1.5–27.3°
*b* = 45.892 (4) Å	µ = 1.10 mm^−^^1^
*c* = 14.8108 (13) Å	*T* = 296 K
β = 92.828 (1)°	Needle, red
*V* = 9320.4 (14) Å^3^	0.38 × 0.16 × 0.12 mm
*Z* = 12	

### Data collection

**Table:**

Bruker SMART CCD diffractometer	21227 independent reflections
Radiation source: fine-focus sealed tube	12089 reflections with *I* > 2σ(*I*)
Graphite monochromator	*R*_int_ = 0.036
φ and ω scans	θ_max_ = 27.5°, θ_min_ = 1.5°
Absorption correction: multi-scan (*SADABS*; Bruker, 2004)	*h* = −17→16
*T*_min_ = 0.806, *T*_max_ = 0.879	*k* = −59→44
57477 measured reflections	*l* = −19→17

### Refinement

**Table:**

Refinement on *F*^2^	Primary atom site location: structure-invariant direct methods
Least-squares matrix: full	Secondary atom site location: difference Fourier map
*R*[*F*^2^ > 2σ(*F*^2^)] = 0.061	Hydrogen site location: inferred from neighbouring sites
*wR*(*F*^2^) = 0.171	H-atom parameters constrained
*S* = 1.01	*w* = 1/[σ^2^(*F*_o_^2^) + (0.0702*P*)^2^ + 9.5182*P*] where *P* = (*F*_o_^2^ + 2*F*_c_^2^)/3
21227 reflections	(Δ/σ)_max_ = 0.002
1090 parameters	Δρ_max_ = 0.56 e Å^−^^3^
0 restraints	Δρ_min_ = −0.46 e Å^−^^3^

### Special details

**Table:**

Geometry. All e.s.d.'s (except the e.s.d. in the dihedral angle between two l.s. planes) are estimated using the full covariance matrix. The cell e.s.d.'s are taken into account individually in the estimation of e.s.d.'s in distances, angles and torsion angles; correlations between e.s.d.'s in cell parameters are only used when they are defined by crystal symmetry. An approximate (isotropic) treatment of cell e.s.d.'s is used for estimating e.s.d.'s involving l.s. planes.
Refinement. Refinement of *F*^2^ against ALL reflections. The weighted *R*-factor *wR* and goodness of fit *S* are based on *F*^2^, conventional *R*-factors *R* are based on *F*, with *F* set to zero for negative *F*^2^. The threshold expression of *F*^2^ > σ(*F*^2^) is used only for calculating *R*-factors(gt) *etc*. and is not relevant to the choice of reflections for refinement. *R*-factors based on *F*^2^ are statistically about twice as large as those based on *F*, and *R*- factors based on ALL data will be even larger.

### Fractional atomic coordinates and isotropic or equivalent isotropic displacement parameters (Å^2^)

**Table:**

	*x*	*y*	*z*	*U*_iso_*/*U*_eq_	
C1	0.9142 (3)	0.21845 (9)	0.8077 (3)	0.0497 (10)	
C2	0.9884 (3)	0.21668 (10)	0.8766 (3)	0.0607 (12)	
C3	1.0680 (4)	0.23459 (11)	0.8786 (4)	0.0715 (14)	
H3	1.1167	0.2326	0.9241	0.086*	
C4	1.0756 (4)	0.25545 (11)	0.8134 (4)	0.0742 (15)	
C5	1.0036 (3)	0.25982 (10)	0.7491 (4)	0.0665 (13)	
H5	1.0087	0.2748	0.7073	0.080*	
C6	0.9210 (3)	0.24161 (9)	0.7455 (3)	0.0507 (10)	
C7	0.8394 (3)	0.24793 (9)	0.6845 (3)	0.0542 (10)	
H7	0.8371	0.2665	0.6592	0.065*	
C8	0.6897 (3)	0.24308 (10)	0.6026 (3)	0.0631 (12)	
H8A	0.6452	0.2276	0.5836	0.076*	
H8B	0.7173	0.2510	0.5488	0.076*	
C9	0.6336 (4)	0.26642 (11)	0.6468 (4)	0.0766 (15)	
H9A	0.6021	0.2584	0.6977	0.115*	
H9B	0.5853	0.2742	0.6043	0.115*	
H9C	0.6772	0.2817	0.6669	0.115*	
C10	0.8351 (3)	0.13983 (9)	0.7865 (3)	0.0531 (11)	
C11	0.8320 (4)	0.12193 (9)	0.8646 (3)	0.0603 (12)	
C12	0.9115 (4)	0.10708 (9)	0.8994 (4)	0.0670 (13)	
H12	0.9066	0.0958	0.9511	0.080*	
C13	0.9992 (4)	0.10889 (10)	0.8578 (4)	0.0646 (13)	
C14	1.0071 (4)	0.12578 (10)	0.7828 (3)	0.0634 (12)	
H14	1.0658	0.1265	0.7542	0.076*	
C15	0.9271 (3)	0.14217 (9)	0.7484 (3)	0.0521 (10)	
C16	0.9403 (3)	0.15995 (10)	0.6715 (3)	0.0599 (12)	
H16	0.9966	0.1567	0.6406	0.072*	
C17	0.9126 (5)	0.19453 (12)	0.5562 (4)	0.0861 (17)	
H17A	0.8981	0.2152	0.5606	0.103*	
H17B	0.9828	0.1926	0.5534	0.103*	
C18	0.8678 (5)	0.1837 (2)	0.4719 (5)	0.134 (3)	
H18A	0.8862	0.1637	0.4636	0.202*	
H18B	0.8893	0.1951	0.4225	0.202*	
H18C	0.7981	0.1850	0.4738	0.202*	
C19	0.6101 (3)	0.16329 (9)	0.6041 (3)	0.0567 (11)	
C20	0.5803 (4)	0.14260 (10)	0.5379 (3)	0.0700 (14)	
C21	0.4917 (5)	0.12901 (12)	0.5376 (5)	0.0893 (18)	
H21	0.4747	0.1154	0.4930	0.107*	
C22	0.4274 (4)	0.13550 (15)	0.6036 (5)	0.0953 (19)	
C23	0.4525 (4)	0.15488 (13)	0.6693 (4)	0.0821 (16)	
H23	0.4085	0.1591	0.7132	0.099*	
C24	0.5432 (3)	0.16860 (10)	0.6724 (3)	0.0622 (12)	
C25	0.5664 (3)	0.18833 (10)	0.7454 (3)	0.0608 (12)	
H25	0.5153	0.1936	0.7810	0.073*	
C26	0.6562 (4)	0.21848 (10)	0.8489 (3)	0.0637 (12)	
H26A	0.5951	0.2289	0.8540	0.076*	
H26B	0.7073	0.2328	0.8424	0.076*	
C27	0.6776 (4)	0.20096 (13)	0.9338 (3)	0.0819 (16)	
H27A	0.6276	0.1866	0.9398	0.123*	
H27B	0.6792	0.2137	0.9853	0.123*	
H27C	0.7396	0.1915	0.9303	0.123*	
C28	0.0809 (3)	0.51848 (9)	0.2276 (3)	0.0485 (10)	
C29	0.0065 (3)	0.53817 (10)	0.1989 (3)	0.0562 (11)	
C30	−0.0558 (3)	0.55071 (11)	0.2575 (4)	0.0664 (13)	
H30	−0.1024	0.5641	0.2364	0.080*	
C31	−0.0488 (4)	0.54327 (11)	0.3475 (4)	0.0690 (13)	
C32	0.0182 (3)	0.52351 (10)	0.3780 (3)	0.0617 (12)	
H32	0.0210	0.5183	0.4388	0.074*	
C33	0.0830 (3)	0.51079 (9)	0.3200 (3)	0.0509 (10)	
C34	0.1529 (3)	0.48985 (9)	0.3574 (3)	0.0547 (11)	
H34	0.1453	0.4842	0.4169	0.066*	
C35	0.2840 (4)	0.45705 (10)	0.3715 (3)	0.0677 (13)	
H35A	0.3096	0.4426	0.3312	0.081*	
H35B	0.2440	0.4470	0.4139	0.081*	
C36	0.3675 (4)	0.47211 (13)	0.4226 (4)	0.0908 (18)	
H36A	0.4058	0.4826	0.3810	0.136*	
H36B	0.4076	0.4579	0.4540	0.136*	
H36C	0.3421	0.4855	0.4655	0.136*	
C37	0.4003 (3)	0.53149 (8)	0.2233 (3)	0.0479 (10)	
C38	0.4670 (3)	0.54566 (9)	0.2850 (3)	0.0575 (11)	
C39	0.5542 (3)	0.55657 (11)	0.2603 (4)	0.0664 (13)	
H39	0.5960	0.5655	0.3032	0.080*	
C40	0.5802 (3)	0.55446 (11)	0.1726 (4)	0.0663 (13)	
C41	0.5194 (3)	0.54094 (10)	0.1090 (3)	0.0590 (11)	
H41	0.5374	0.5396	0.0494	0.071*	
C42	0.4308 (3)	0.52934 (9)	0.1337 (3)	0.0528 (10)	
C43	0.3669 (3)	0.51664 (9)	0.0638 (3)	0.0532 (10)	
H43	0.3817	0.5203	0.0043	0.064*	
C44	0.2279 (4)	0.49301 (12)	−0.0030 (3)	0.0716 (14)	
H44A	0.2590	0.4774	−0.0348	0.086*	
H44B	0.1677	0.4853	0.0190	0.086*	
C45	0.2027 (6)	0.51563 (17)	−0.0677 (5)	0.131 (3)	
H45A	0.1975	0.5339	−0.0369	0.197*	
H45B	0.1415	0.5111	−0.0985	0.197*	
H45C	0.2525	0.5170	−0.1109	0.197*	
C46	0.4197 (3)	0.45143 (9)	0.1672 (3)	0.0512 (10)	
C47	0.5224 (3)	0.44814 (10)	0.1663 (3)	0.0631 (12)	
C48	0.5638 (4)	0.42690 (11)	0.1153 (4)	0.0728 (14)	
H48	0.6314	0.4254	0.1149	0.087*	
C49	0.5060 (4)	0.40779 (11)	0.0649 (4)	0.0679 (13)	
C50	0.4075 (4)	0.40898 (10)	0.0676 (3)	0.0663 (13)	
H50	0.3692	0.3953	0.0359	0.080*	
C51	0.3634 (3)	0.43063 (9)	0.1178 (3)	0.0559 (11)	
C52	0.2587 (3)	0.43080 (10)	0.1211 (3)	0.0573 (11)	
H52	0.2262	0.4144	0.0981	0.069*	
C53	0.0992 (3)	0.44455 (11)	0.1522 (4)	0.0697 (13)	
H53A	0.0637	0.4626	0.1595	0.084*	
H53B	0.0785	0.4363	0.0941	0.084*	
C54	0.0736 (5)	0.42406 (16)	0.2244 (5)	0.114 (2)	
H54A	0.1097	0.4063	0.2186	0.171*	
H54B	0.0050	0.4199	0.2191	0.171*	
H54C	0.0896	0.4327	0.2823	0.171*	
C55	0.8113 (3)	0.31424 (9)	0.5174 (4)	0.0593 (12)	
C56	0.8568 (4)	0.29457 (10)	0.4594 (4)	0.0708 (14)	
C57	0.8276 (4)	0.29227 (12)	0.3677 (4)	0.0786 (16)	
H57	0.8583	0.2790	0.3313	0.094*	
C58	0.7541 (5)	0.30948 (14)	0.3314 (4)	0.0826 (16)	
C59	0.7063 (4)	0.32798 (12)	0.3856 (4)	0.0756 (14)	
H59	0.6542	0.3389	0.3615	0.091*	
C60	0.7346 (4)	0.33068 (10)	0.4770 (3)	0.0637 (12)	
C61	0.6745 (3)	0.34863 (9)	0.5313 (3)	0.0598 (12)	
H61	0.6145	0.3545	0.5053	0.072*	
C62	0.6180 (4)	0.37165 (11)	0.6619 (4)	0.0717 (14)	
H62A	0.6364	0.3708	0.7259	0.086*	
H62B	0.6157	0.3920	0.6446	0.086*	
C63	0.5189 (4)	0.35949 (17)	0.6485 (5)	0.120 (3)	
H63A	0.4934	0.3641	0.5887	0.180*	
H63B	0.4775	0.3677	0.6921	0.180*	
H63C	0.5214	0.3387	0.6559	0.180*	
C64	0.8613 (3)	0.39335 (10)	0.5342 (3)	0.0593 (12)	
C65	0.8834 (4)	0.39986 (11)	0.4447 (4)	0.0702 (14)	
C66	0.8610 (4)	0.42587 (12)	0.4045 (4)	0.0728 (14)	
H66	0.8760	0.4292	0.3449	0.087*	
C67	0.8154 (4)	0.44733 (11)	0.4536 (4)	0.0684 (13)	
C68	0.7953 (3)	0.44267 (10)	0.5411 (3)	0.0626 (12)	
H68	0.7662	0.4574	0.5737	0.075*	
C69	0.8177 (3)	0.41599 (9)	0.5829 (3)	0.0558 (11)	
C70	0.8075 (4)	0.41329 (10)	0.6794 (3)	0.0636 (12)	
H70	0.7930	0.4302	0.7106	0.076*	
C71	0.8172 (5)	0.39182 (11)	0.8255 (4)	0.0868 (18)	
H71A	0.8845	0.3935	0.8478	0.104*	
H71B	0.7927	0.3735	0.8480	0.104*	
C72	0.7614 (5)	0.41564 (15)	0.8664 (4)	0.098 (2)	
H72A	0.7870	0.4341	0.8484	0.148*	
H72B	0.7669	0.4140	0.9311	0.148*	
H72C	0.6940	0.4142	0.8463	0.148*	
C73	0.7937 (3)	0.31835 (9)	0.8286 (3)	0.0571 (11)	
C74	0.7299 (4)	0.30417 (11)	0.8882 (4)	0.0681 (13)	
C75	0.7628 (4)	0.28850 (11)	0.9608 (4)	0.0729 (14)	
H75	0.7187	0.2792	0.9968	0.088*	
C76	0.8618 (4)	0.28632 (11)	0.9817 (4)	0.0735 (14)	
C77	0.9266 (4)	0.30007 (10)	0.9292 (4)	0.0689 (13)	
H77	0.9930	0.2990	0.9444	0.083*	
C78	0.8939 (3)	0.31586 (10)	0.8522 (3)	0.0605 (12)	
C79	0.9658 (4)	0.33031 (12)	0.8009 (4)	0.0731 (14)	
H79	1.0302	0.3285	0.8226	0.088*	
C80	1.0417 (5)	0.3608 (2)	0.6948 (5)	0.118 (3)	
H80A	1.0238	0.3796	0.6687	0.141*	
H80B	1.0895	0.3640	0.7443	0.141*	
C81	1.0799 (6)	0.34268 (19)	0.6293 (7)	0.144 (3)	
H81A	1.1042	0.3251	0.6574	0.217*	
H81B	1.1322	0.3525	0.6012	0.217*	
H81C	1.0297	0.3380	0.5843	0.217*	
Cl1	0.97389 (10)	0.19083 (3)	0.96043 (9)	0.0771 (4)	
Cl2	1.17926 (11)	0.27773 (4)	0.81744 (15)	0.1178 (7)	
Cl3	0.72211 (11)	0.11913 (3)	0.91688 (11)	0.0856 (4)	
Cl4	1.10024 (12)	0.08948 (3)	0.90073 (12)	0.0970 (5)	
Cl5	0.66061 (12)	0.13391 (3)	0.45487 (10)	0.0903 (4)	
Cl6	0.31476 (17)	0.11843 (7)	0.6022 (2)	0.1816 (12)	
Cl7	−0.00402 (10)	0.54739 (3)	0.08487 (9)	0.0776 (4)	
Cl8	−0.12587 (14)	0.56038 (4)	0.42125 (13)	0.1113 (6)	
Cl9	0.43621 (10)	0.54929 (3)	0.39664 (9)	0.0789 (4)	
Cl10	0.69115 (11)	0.56965 (4)	0.14323 (12)	0.0998 (5)	
Cl11	0.59460 (10)	0.47149 (3)	0.23061 (11)	0.0897 (4)	
Cl12	0.56047 (13)	0.38168 (3)	−0.00201 (13)	0.1067 (6)	
Cl13	0.95085 (11)	0.27305 (3)	0.50221 (12)	0.0951 (5)	
Cl14	0.71771 (16)	0.30583 (5)	0.21810 (12)	0.1239 (7)	
Cl15	0.94446 (14)	0.37346 (4)	0.38532 (12)	0.1068 (6)	
Cl16	0.78918 (13)	0.48081 (3)	0.40164 (11)	0.0949 (5)	
Cl17	0.60526 (11)	0.30817 (4)	0.86484 (12)	0.0960 (5)	
Cl18	0.90338 (14)	0.26587 (4)	1.07422 (11)	0.1053 (5)	
Co1	0.76546 (4)	0.190541 (12)	0.70306 (4)	0.04715 (15)	
Co2	0.25845 (4)	0.488212 (12)	0.19652 (4)	0.04676 (15)	
Co3	0.82477 (4)	0.351743 (13)	0.66808 (4)	0.05682 (17)	
N1	0.7693 (2)	0.23057 (7)	0.6614 (2)	0.0494 (8)	
N2	0.8826 (3)	0.18009 (8)	0.6399 (2)	0.0553 (9)	
N3	0.6495 (3)	0.19946 (7)	0.7670 (2)	0.0525 (9)	
N4	0.2231 (3)	0.47832 (7)	0.3181 (2)	0.0506 (8)	
N5	0.2917 (3)	0.50076 (7)	0.0761 (2)	0.0495 (8)	
N6	0.2054 (2)	0.45112 (8)	0.1524 (2)	0.0513 (8)	
N7	0.6958 (3)	0.35731 (7)	0.6118 (3)	0.0573 (9)	
N8	0.8165 (3)	0.38984 (8)	0.7258 (3)	0.0594 (10)	
N9	0.9513 (3)	0.34534 (10)	0.7287 (3)	0.0742 (12)	
O1	0.8428 (2)	0.19994 (6)	0.80680 (19)	0.0505 (7)	
O2	0.7572 (2)	0.15262 (6)	0.7540 (2)	0.0540 (7)	
O3	0.6929 (2)	0.17702 (7)	0.5992 (2)	0.0593 (8)	
O4	0.1390 (2)	0.50795 (6)	0.16963 (19)	0.0519 (7)	
O5	0.3153 (2)	0.52279 (6)	0.24818 (19)	0.0512 (7)	
O6	0.3836 (2)	0.47185 (6)	0.2159 (2)	0.0537 (7)	
O7	0.8379 (2)	0.31547 (6)	0.6040 (2)	0.0617 (8)	
O8	0.8834 (2)	0.36810 (7)	0.5685 (2)	0.0629 (8)	
O9	0.7572 (2)	0.33274 (7)	0.7592 (2)	0.0622 (8)	

### Atomic displacement parameters (Å^2^)

**Table:**

	*U*^11^	*U*^22^	*U*^33^	*U*^12^	*U*^13^	*U*^23^
C1	0.049 (2)	0.044 (2)	0.056 (3)	0.0072 (19)	0.000 (2)	−0.0075 (19)
C2	0.062 (3)	0.054 (3)	0.064 (3)	0.008 (2)	−0.009 (2)	−0.012 (2)
C3	0.060 (3)	0.066 (3)	0.086 (4)	0.009 (2)	−0.028 (3)	−0.015 (3)
C4	0.058 (3)	0.053 (3)	0.109 (4)	−0.004 (2)	−0.018 (3)	−0.004 (3)
C5	0.059 (3)	0.050 (3)	0.090 (4)	−0.003 (2)	−0.005 (3)	0.000 (2)
C6	0.047 (2)	0.045 (2)	0.059 (3)	0.0022 (18)	−0.001 (2)	−0.007 (2)
C7	0.058 (3)	0.046 (2)	0.059 (3)	−0.001 (2)	0.000 (2)	0.004 (2)
C8	0.060 (3)	0.062 (3)	0.065 (3)	0.003 (2)	−0.015 (2)	0.005 (2)
C9	0.066 (3)	0.068 (3)	0.094 (4)	0.010 (3)	−0.015 (3)	0.001 (3)
C10	0.064 (3)	0.036 (2)	0.059 (3)	−0.001 (2)	−0.002 (2)	−0.0058 (19)
C11	0.077 (3)	0.039 (2)	0.065 (3)	0.001 (2)	0.009 (2)	−0.004 (2)
C12	0.090 (4)	0.041 (2)	0.069 (3)	0.001 (2)	−0.008 (3)	0.002 (2)
C13	0.074 (3)	0.045 (2)	0.073 (3)	0.010 (2)	−0.015 (3)	−0.006 (2)
C14	0.060 (3)	0.054 (3)	0.076 (3)	0.006 (2)	0.001 (2)	−0.015 (2)
C15	0.057 (3)	0.046 (2)	0.054 (3)	0.004 (2)	0.004 (2)	−0.0085 (19)
C16	0.058 (3)	0.058 (3)	0.064 (3)	0.002 (2)	0.008 (2)	−0.011 (2)
C17	0.131 (5)	0.064 (3)	0.066 (4)	0.005 (3)	0.034 (4)	0.005 (3)
C18	0.101 (5)	0.220 (9)	0.083 (5)	−0.013 (6)	0.003 (4)	0.053 (6)
C19	0.063 (3)	0.050 (2)	0.055 (3)	0.001 (2)	−0.015 (2)	0.005 (2)
C20	0.090 (4)	0.053 (3)	0.065 (3)	0.003 (3)	−0.022 (3)	−0.002 (2)
C21	0.094 (4)	0.071 (4)	0.100 (5)	−0.014 (3)	−0.033 (4)	−0.017 (3)
C22	0.071 (4)	0.106 (5)	0.106 (5)	−0.032 (3)	−0.021 (4)	−0.008 (4)
C23	0.061 (3)	0.092 (4)	0.092 (4)	−0.014 (3)	−0.009 (3)	−0.001 (3)
C24	0.061 (3)	0.062 (3)	0.062 (3)	−0.008 (2)	−0.011 (2)	0.004 (2)
C25	0.050 (3)	0.065 (3)	0.068 (3)	0.006 (2)	0.004 (2)	0.003 (2)
C26	0.063 (3)	0.062 (3)	0.067 (3)	0.006 (2)	0.010 (2)	−0.012 (2)
C27	0.090 (4)	0.102 (4)	0.054 (3)	0.007 (3)	0.012 (3)	−0.005 (3)
C28	0.045 (2)	0.044 (2)	0.056 (3)	−0.0069 (18)	−0.005 (2)	0.0045 (19)
C29	0.049 (3)	0.056 (3)	0.062 (3)	−0.006 (2)	−0.008 (2)	0.004 (2)
C30	0.051 (3)	0.061 (3)	0.088 (4)	0.004 (2)	0.003 (3)	0.003 (3)
C31	0.057 (3)	0.064 (3)	0.087 (4)	0.004 (2)	0.017 (3)	0.000 (3)
C32	0.062 (3)	0.063 (3)	0.061 (3)	−0.011 (2)	0.007 (2)	0.005 (2)
C33	0.049 (2)	0.046 (2)	0.057 (3)	−0.0050 (19)	−0.002 (2)	0.002 (2)
C34	0.067 (3)	0.050 (2)	0.047 (3)	−0.004 (2)	0.003 (2)	0.0059 (19)
C35	0.083 (3)	0.059 (3)	0.060 (3)	0.015 (3)	−0.005 (3)	0.014 (2)
C36	0.104 (4)	0.084 (4)	0.080 (4)	0.029 (3)	−0.033 (3)	0.003 (3)
C37	0.051 (2)	0.040 (2)	0.053 (3)	0.0006 (18)	−0.008 (2)	0.0051 (18)
C38	0.062 (3)	0.053 (3)	0.056 (3)	0.002 (2)	−0.014 (2)	−0.006 (2)
C39	0.053 (3)	0.071 (3)	0.073 (4)	−0.008 (2)	−0.016 (2)	−0.006 (3)
C40	0.053 (3)	0.070 (3)	0.075 (4)	−0.011 (2)	−0.005 (2)	0.003 (3)
C41	0.054 (3)	0.062 (3)	0.061 (3)	−0.009 (2)	−0.003 (2)	0.007 (2)
C42	0.052 (2)	0.048 (2)	0.057 (3)	−0.0083 (19)	−0.010 (2)	0.0055 (19)
C43	0.056 (3)	0.054 (3)	0.049 (3)	−0.008 (2)	−0.002 (2)	0.0098 (19)
C44	0.075 (3)	0.082 (3)	0.056 (3)	−0.016 (3)	−0.018 (2)	−0.002 (3)
C45	0.144 (6)	0.142 (6)	0.099 (5)	−0.059 (5)	−0.073 (5)	0.047 (5)
C46	0.055 (3)	0.049 (2)	0.049 (3)	0.004 (2)	0.000 (2)	0.010 (2)
C47	0.057 (3)	0.055 (3)	0.077 (3)	−0.003 (2)	0.000 (2)	0.017 (2)
C48	0.060 (3)	0.068 (3)	0.092 (4)	0.013 (3)	0.018 (3)	0.023 (3)
C49	0.071 (3)	0.057 (3)	0.078 (4)	0.008 (3)	0.023 (3)	0.011 (3)
C50	0.077 (3)	0.047 (3)	0.076 (3)	0.004 (2)	0.013 (3)	0.004 (2)
C51	0.060 (3)	0.049 (2)	0.059 (3)	−0.002 (2)	0.006 (2)	0.007 (2)
C52	0.064 (3)	0.045 (2)	0.063 (3)	−0.010 (2)	−0.003 (2)	−0.002 (2)
C53	0.058 (3)	0.067 (3)	0.083 (4)	−0.014 (2)	0.003 (3)	−0.004 (3)
C54	0.107 (5)	0.138 (6)	0.098 (5)	−0.053 (5)	0.020 (4)	−0.001 (4)
C55	0.065 (3)	0.041 (2)	0.073 (3)	−0.010 (2)	0.019 (3)	0.002 (2)
C56	0.071 (3)	0.048 (3)	0.096 (4)	−0.010 (2)	0.027 (3)	0.006 (3)
C57	0.085 (4)	0.064 (3)	0.090 (4)	−0.020 (3)	0.034 (3)	−0.014 (3)
C58	0.091 (4)	0.087 (4)	0.072 (4)	−0.019 (3)	0.021 (3)	−0.007 (3)
C59	0.081 (4)	0.077 (4)	0.069 (4)	−0.011 (3)	0.004 (3)	−0.008 (3)
C60	0.071 (3)	0.047 (2)	0.073 (3)	−0.007 (2)	0.003 (3)	−0.002 (2)
C61	0.061 (3)	0.046 (2)	0.072 (3)	−0.010 (2)	−0.003 (2)	−0.002 (2)
C62	0.072 (3)	0.060 (3)	0.083 (4)	0.005 (3)	0.006 (3)	−0.007 (3)
C63	0.072 (4)	0.146 (7)	0.145 (7)	−0.008 (4)	0.025 (4)	−0.052 (5)
C64	0.057 (3)	0.053 (3)	0.068 (3)	−0.016 (2)	0.005 (2)	0.002 (2)
C65	0.075 (3)	0.059 (3)	0.077 (4)	−0.019 (3)	0.017 (3)	−0.002 (3)
C66	0.078 (3)	0.072 (3)	0.069 (3)	−0.026 (3)	0.006 (3)	0.005 (3)
C67	0.072 (3)	0.055 (3)	0.076 (4)	−0.022 (2)	−0.010 (3)	0.006 (3)
C68	0.069 (3)	0.049 (3)	0.069 (3)	−0.018 (2)	−0.001 (2)	−0.004 (2)
C69	0.063 (3)	0.044 (2)	0.060 (3)	−0.015 (2)	−0.001 (2)	0.001 (2)
C70	0.075 (3)	0.047 (3)	0.068 (3)	−0.011 (2)	−0.005 (2)	−0.007 (2)
C71	0.138 (5)	0.058 (3)	0.062 (3)	0.004 (3)	−0.018 (3)	−0.004 (2)
C72	0.111 (5)	0.114 (5)	0.071 (4)	0.018 (4)	0.016 (3)	0.010 (3)
C73	0.067 (3)	0.040 (2)	0.064 (3)	0.000 (2)	0.003 (2)	−0.003 (2)
C74	0.066 (3)	0.059 (3)	0.080 (4)	0.010 (2)	0.015 (3)	0.000 (3)
C75	0.087 (4)	0.059 (3)	0.074 (4)	0.005 (3)	0.017 (3)	0.006 (3)
C76	0.094 (4)	0.060 (3)	0.067 (3)	0.013 (3)	0.002 (3)	0.002 (2)
C77	0.071 (3)	0.057 (3)	0.078 (4)	0.008 (2)	−0.006 (3)	−0.001 (3)
C78	0.060 (3)	0.050 (3)	0.071 (3)	0.006 (2)	0.000 (2)	−0.004 (2)
C79	0.056 (3)	0.080 (4)	0.082 (4)	0.002 (3)	0.000 (3)	0.012 (3)
C80	0.076 (4)	0.173 (8)	0.104 (6)	0.010 (5)	0.000 (4)	0.017 (5)
C81	0.120 (7)	0.127 (7)	0.188 (10)	−0.014 (5)	0.026 (6)	0.013 (6)
Cl1	0.0859 (9)	0.0742 (8)	0.0685 (8)	0.0081 (7)	−0.0236 (7)	0.0079 (6)
Cl2	0.0704 (9)	0.0909 (11)	0.188 (2)	−0.0264 (8)	−0.0382 (11)	0.0116 (11)
Cl3	0.0955 (10)	0.0677 (8)	0.0957 (11)	−0.0033 (7)	0.0273 (8)	0.0176 (7)
Cl4	0.0973 (11)	0.0746 (9)	0.1155 (13)	0.0278 (8)	−0.0304 (9)	0.0015 (8)
Cl5	0.1152 (12)	0.0840 (9)	0.0701 (9)	0.0075 (8)	−0.0101 (8)	−0.0217 (7)
Cl6	0.1069 (15)	0.228 (3)	0.209 (3)	−0.0955 (18)	0.0015 (16)	−0.071 (2)
Cl7	0.0717 (8)	0.0898 (9)	0.0691 (8)	0.0089 (7)	−0.0199 (6)	0.0152 (7)
Cl8	0.1137 (13)	0.1053 (12)	0.1193 (14)	0.0356 (10)	0.0512 (11)	0.0074 (10)
Cl9	0.0805 (9)	0.0941 (10)	0.0609 (8)	−0.0050 (7)	−0.0095 (6)	−0.0164 (7)
Cl10	0.0663 (9)	0.1287 (13)	0.1037 (12)	−0.0392 (9)	−0.0035 (8)	−0.0040 (10)
Cl11	0.0565 (7)	0.0960 (10)	0.1142 (12)	−0.0061 (7)	−0.0186 (7)	0.0025 (9)
Cl12	0.1127 (12)	0.0775 (10)	0.1351 (15)	0.0153 (9)	0.0608 (11)	−0.0008 (9)
Cl13	0.0921 (10)	0.0729 (9)	0.1245 (13)	0.0169 (8)	0.0483 (9)	0.0118 (8)
Cl14	0.1443 (16)	0.1519 (17)	0.0768 (11)	−0.0220 (13)	0.0176 (11)	−0.0270 (11)
Cl15	0.1351 (14)	0.0879 (11)	0.1022 (12)	−0.0030 (10)	0.0562 (11)	−0.0051 (9)
Cl16	0.1299 (13)	0.0674 (8)	0.0855 (10)	−0.0149 (8)	−0.0131 (9)	0.0184 (7)
Cl17	0.0665 (8)	0.1184 (13)	0.1049 (12)	0.0024 (8)	0.0207 (8)	0.0178 (9)
Cl18	0.1338 (14)	0.0940 (11)	0.0876 (11)	0.0307 (10)	0.0012 (10)	0.0232 (9)
Co1	0.0507 (3)	0.0425 (3)	0.0480 (3)	−0.0004 (2)	0.0000 (2)	−0.0009 (2)
Co2	0.0479 (3)	0.0439 (3)	0.0477 (3)	−0.0023 (2)	−0.0048 (2)	0.0038 (2)
Co3	0.0571 (4)	0.0457 (3)	0.0675 (4)	−0.0059 (3)	0.0018 (3)	0.0013 (3)
N1	0.051 (2)	0.0453 (19)	0.052 (2)	0.0009 (16)	−0.0033 (16)	0.0033 (15)
N2	0.066 (2)	0.048 (2)	0.052 (2)	−0.0027 (18)	0.0126 (18)	0.0005 (16)
N3	0.057 (2)	0.049 (2)	0.052 (2)	0.0029 (17)	0.0036 (17)	−0.0043 (16)
N4	0.054 (2)	0.0452 (19)	0.051 (2)	0.0014 (16)	−0.0086 (17)	0.0071 (16)
N5	0.054 (2)	0.0472 (19)	0.046 (2)	−0.0050 (16)	−0.0044 (16)	−0.0015 (15)
N6	0.0456 (19)	0.050 (2)	0.058 (2)	−0.0078 (16)	−0.0008 (16)	0.0042 (17)
N7	0.065 (2)	0.0406 (19)	0.067 (3)	−0.0056 (17)	0.0034 (19)	−0.0021 (17)
N8	0.071 (3)	0.049 (2)	0.058 (2)	−0.0109 (18)	−0.0051 (19)	−0.0011 (18)
N9	0.053 (2)	0.082 (3)	0.088 (3)	−0.016 (2)	0.005 (2)	0.014 (2)
O1	0.0524 (17)	0.0474 (16)	0.0511 (17)	−0.0011 (13)	−0.0042 (13)	−0.0013 (13)
O2	0.0540 (17)	0.0426 (16)	0.066 (2)	−0.0003 (13)	0.0043 (15)	0.0007 (13)
O3	0.065 (2)	0.0622 (19)	0.0502 (18)	−0.0092 (16)	0.0016 (15)	−0.0059 (14)
O4	0.0483 (16)	0.0551 (17)	0.0516 (17)	0.0045 (13)	−0.0041 (14)	0.0071 (13)
O5	0.0563 (18)	0.0468 (16)	0.0499 (17)	−0.0063 (13)	−0.0037 (13)	0.0013 (13)
O6	0.0489 (16)	0.0528 (17)	0.0585 (18)	−0.0008 (13)	−0.0073 (14)	0.0028 (14)
O7	0.0642 (19)	0.0414 (16)	0.080 (2)	0.0002 (14)	0.0069 (17)	0.0025 (15)
O8	0.070 (2)	0.0484 (18)	0.071 (2)	−0.0076 (15)	0.0129 (16)	0.0016 (15)
O9	0.0581 (19)	0.0582 (19)	0.071 (2)	−0.0024 (15)	0.0063 (16)	0.0068 (16)

### Geometric parameters (Å, º)

**Table:**

C1—O1	1.297 (5)	C45—H45C	0.9600
C1—C2	1.409 (6)	C46—O6	1.296 (5)
C1—C6	1.413 (6)	C46—C51	1.411 (6)
C2—C3	1.366 (7)	C46—C47	1.418 (6)
C2—Cl1	1.735 (5)	C47—C48	1.373 (7)
C3—C4	1.368 (7)	C47—Cl11	1.716 (5)
C3—H3	0.9300	C48—C49	1.378 (7)
C4—C5	1.353 (7)	C48—H48	0.9300
C4—Cl2	1.751 (5)	C49—C50	1.355 (7)
C5—C6	1.407 (6)	C49—Cl12	1.746 (5)
C5—H5	0.9300	C50—C51	1.397 (6)
C6—C7	1.433 (6)	C50—H50	0.9300
C7—N1	1.283 (5)	C51—C52	1.439 (6)
C7—H7	0.9300	C52—N6	1.286 (5)
C8—N1	1.479 (5)	C52—H52	0.9300
C8—C9	1.490 (7)	C53—C54	1.479 (8)
C8—H8A	0.9700	C53—N6	1.489 (6)
C8—H8B	0.9700	C53—H53A	0.9700
C9—H9A	0.9600	C53—H53B	0.9700
C9—H9B	0.9600	C54—H54A	0.9600
C9—H9C	0.9600	C54—H54B	0.9600
C10—O2	1.291 (5)	C54—H54C	0.9600
C10—C15	1.413 (6)	C55—O7	1.317 (6)
C10—C11	1.422 (6)	C55—C60	1.405 (7)
C11—C12	1.366 (7)	C55—C56	1.413 (7)
C11—Cl3	1.734 (5)	C56—C57	1.400 (8)
C12—C13	1.381 (7)	C56—Cl13	1.722 (6)
C12—H12	0.9300	C57—C58	1.370 (8)
C13—C14	1.364 (7)	C57—H57	0.9300
C13—Cl4	1.742 (5)	C58—C59	1.360 (8)
C14—C15	1.405 (6)	C58—Cl14	1.735 (6)
C14—H14	0.9300	C59—C60	1.396 (7)
C15—C16	1.419 (6)	C59—H59	0.9300
C16—N2	1.290 (6)	C60—C61	1.439 (7)
C16—H16	0.9300	C61—N7	1.278 (6)
C17—C18	1.453 (9)	C61—H61	0.9300
C17—N2	1.483 (6)	C62—C63	1.475 (8)
C17—H17A	0.9700	C62—N7	1.484 (6)
C17—H17B	0.9700	C62—H62A	0.9700
C18—H18A	0.9600	C62—H62B	0.9700
C18—H18B	0.9600	C63—H63A	0.9600
C18—H18C	0.9600	C63—H63B	0.9600
C19—O3	1.304 (5)	C63—H63C	0.9600
C19—C20	1.410 (6)	C64—O8	1.295 (5)
C19—C24	1.421 (7)	C64—C65	1.407 (7)
C20—C21	1.368 (8)	C64—C69	1.414 (6)
C20—Cl5	1.738 (6)	C65—C66	1.362 (7)
C21—C22	1.381 (9)	C65—Cl15	1.737 (5)
C21—H21	0.9300	C66—C67	1.391 (7)
C22—C23	1.350 (8)	C66—H66	0.9300
C22—Cl6	1.733 (6)	C67—C68	1.356 (7)
C23—C24	1.395 (7)	C67—Cl16	1.747 (5)
C23—H23	0.9300	C68—C69	1.399 (6)
C24—C25	1.434 (7)	C68—H68	0.9300
C25—N3	1.276 (5)	C69—C70	1.448 (6)
C25—H25	0.9300	C70—N8	1.280 (6)
C26—N3	1.493 (6)	C70—H70	0.9300
C26—C27	1.509 (7)	C71—N8	1.479 (6)
C26—H26A	0.9700	C71—C72	1.482 (8)
C26—H26B	0.9700	C71—H71A	0.9700
C27—H27A	0.9600	C71—H71B	0.9700
C27—H27B	0.9600	C72—H72A	0.9600
C27—H27C	0.9600	C72—H72B	0.9600
C28—O4	1.294 (5)	C72—H72C	0.9600
C28—C33	1.411 (6)	C73—O9	1.301 (5)
C28—C29	1.413 (6)	C73—C78	1.408 (6)
C29—C30	1.375 (7)	C73—C74	1.431 (7)
C29—Cl7	1.740 (5)	C74—C75	1.353 (7)
C30—C31	1.374 (7)	C74—Cl17	1.739 (5)
C30—H30	0.9300	C75—C76	1.383 (7)
C31—C32	1.353 (7)	C75—H75	0.9300
C31—Cl8	1.745 (5)	C76—C77	1.365 (7)
C32—C33	1.396 (6)	C76—Cl18	1.734 (5)
C32—H32	0.9300	C77—C78	1.405 (7)
C33—C34	1.448 (6)	C77—H77	0.9300
C34—N4	1.266 (5)	C78—C79	1.436 (7)
C34—H34	0.9300	C79—N9	1.279 (6)
C35—N4	1.487 (5)	C79—H79	0.9300
C35—C36	1.510 (7)	C80—C81	1.401 (10)
C35—H35A	0.9700	C80—N9	1.536 (8)
C35—H35B	0.9700	C80—H80A	0.9700
C36—H36A	0.9600	C80—H80B	0.9700
C36—H36B	0.9600	C81—H81A	0.9600
C36—H36C	0.9600	C81—H81B	0.9600
C37—O5	1.304 (5)	C81—H81C	0.9600
C37—C42	1.415 (6)	Co1—O1	1.874 (3)
C37—C38	1.418 (6)	Co1—O3	1.895 (3)
C38—C39	1.364 (7)	Co1—O2	1.902 (3)
C38—Cl9	1.735 (5)	Co1—N3	1.937 (4)
C39—C40	1.368 (7)	Co1—N1	1.939 (3)
C39—H39	0.9300	Co1—N2	1.959 (4)
C40—C41	1.375 (6)	Co2—O6	1.884 (3)
C40—Cl10	1.750 (5)	Co2—O4	1.899 (3)
C41—C42	1.393 (6)	Co2—O5	1.912 (3)
C41—H41	0.9300	Co2—N4	1.943 (4)
C42—C43	1.446 (6)	Co2—N5	1.950 (3)
C43—N5	1.283 (5)	Co2—N6	1.952 (3)
C43—H43	0.9300	Co3—O8	1.872 (3)
C44—C45	1.444 (7)	Co3—O9	1.888 (3)
C44—N5	1.472 (5)	Co3—O7	1.929 (3)
C44—H44A	0.9700	Co3—N7	1.936 (4)
C44—H44B	0.9700	Co3—N9	1.939 (4)
C45—H45A	0.9600	Co3—N8	1.952 (4)
C45—H45B	0.9600		
			
O1—C1—C2	119.3 (4)	C54—C53—H53B	108.9
O1—C1—C6	124.1 (4)	N6—C53—H53B	108.9
C2—C1—C6	116.6 (4)	H53A—C53—H53B	107.7
C3—C2—C1	122.0 (5)	C53—C54—H54A	109.5
C3—C2—Cl1	121.1 (4)	C53—C54—H54B	109.5
C1—C2—Cl1	116.9 (4)	H54A—C54—H54B	109.5
C2—C3—C4	119.6 (4)	C53—C54—H54C	109.5
C2—C3—H3	120.2	H54A—C54—H54C	109.5
C4—C3—H3	120.2	H54B—C54—H54C	109.5
C5—C4—C3	121.5 (5)	O7—C55—C60	123.9 (4)
C5—C4—Cl2	120.1 (4)	O7—C55—C56	120.7 (5)
C3—C4—Cl2	118.3 (4)	C60—C55—C56	115.4 (5)
C4—C5—C6	119.8 (5)	C57—C56—C55	121.6 (5)
C4—C5—H5	120.1	C57—C56—Cl13	119.2 (4)
C6—C5—H5	120.1	C55—C56—Cl13	119.2 (5)
C5—C6—C1	120.1 (4)	C58—C57—C56	120.5 (5)
C5—C6—C7	120.5 (4)	C58—C57—H57	119.8
C1—C6—C7	119.2 (4)	C56—C57—H57	119.8
N1—C7—C6	126.5 (4)	C59—C58—C57	119.7 (6)
N1—C7—H7	116.7	C59—C58—Cl14	120.4 (5)
C6—C7—H7	116.7	C57—C58—Cl14	119.7 (5)
N1—C8—C9	113.8 (4)	C58—C59—C60	120.6 (6)
N1—C8—H8A	108.8	C58—C59—H59	119.7
C9—C8—H8A	108.8	C60—C59—H59	119.7
N1—C8—H8B	108.8	C59—C60—C55	122.2 (5)
C9—C8—H8B	108.8	C59—C60—C61	116.9 (5)
H8A—C8—H8B	107.7	C55—C60—C61	120.5 (5)
C8—C9—H9A	109.5	N7—C61—C60	126.0 (5)
C8—C9—H9B	109.5	N7—C61—H61	117.0
H9A—C9—H9B	109.5	C60—C61—H61	117.0
C8—C9—H9C	109.5	C63—C62—N7	116.6 (4)
H9A—C9—H9C	109.5	C63—C62—H62A	108.1
H9B—C9—H9C	109.5	N7—C62—H62A	108.2
O2—C10—C15	123.9 (4)	C63—C62—H62B	108.2
O2—C10—C11	120.5 (4)	N7—C62—H62B	108.2
C15—C10—C11	115.6 (4)	H62A—C62—H62B	107.3
C12—C11—C10	122.7 (5)	C62—C63—H63A	109.5
C12—C11—Cl3	119.4 (4)	C62—C63—H63B	109.5
C10—C11—Cl3	117.9 (4)	H63A—C63—H63B	109.5
C11—C12—C13	120.0 (5)	C62—C63—H63C	109.5
C11—C12—H12	120.0	H63A—C63—H63C	109.5
C13—C12—H12	120.0	H63B—C63—H63C	109.5
C14—C13—C12	120.2 (4)	O8—C64—C65	120.2 (4)
C14—C13—Cl4	119.6 (4)	O8—C64—C69	123.7 (4)
C12—C13—Cl4	120.2 (4)	C65—C64—C69	116.1 (4)
C13—C14—C15	120.5 (5)	C66—C65—C64	123.0 (5)
C13—C14—H14	119.8	C66—C65—Cl15	119.6 (4)
C15—C14—H14	119.8	C64—C65—Cl15	117.5 (4)
C14—C15—C10	120.8 (4)	C65—C66—C67	119.3 (5)
C14—C15—C16	118.1 (4)	C65—C66—H66	120.4
C10—C15—C16	121.0 (4)	C67—C66—H66	120.3
N2—C16—C15	127.1 (4)	C68—C67—C66	120.4 (5)
N2—C16—H16	116.5	C68—C67—Cl16	120.8 (4)
C15—C16—H16	116.5	C66—C67—Cl16	118.7 (4)
C18—C17—N2	116.2 (5)	C67—C68—C69	120.6 (5)
C18—C17—H17A	108.2	C67—C68—H68	119.7
N2—C17—H17A	108.2	C69—C68—H68	119.7
C18—C17—H17B	108.2	C68—C69—C64	120.5 (4)
N2—C17—H17B	108.2	C68—C69—C70	118.7 (4)
H17A—C17—H17B	107.4	C64—C69—C70	120.3 (4)
C17—C18—H18A	109.5	N8—C70—C69	126.2 (4)
C17—C18—H18B	109.5	N8—C70—H70	116.9
H18A—C18—H18B	109.5	C69—C70—H70	116.9
C17—C18—H18C	109.5	N8—C71—C72	118.4 (5)
H18A—C18—H18C	109.5	N8—C71—H71A	107.7
H18B—C18—H18C	109.5	C72—C71—H71A	107.7
O3—C19—C20	120.7 (5)	N8—C71—H71B	107.7
O3—C19—C24	123.5 (4)	C72—C71—H71B	107.7
C20—C19—C24	115.8 (5)	H71A—C71—H71B	107.1
C21—C20—C19	122.5 (5)	C71—C72—H72A	109.5
C21—C20—Cl5	119.2 (4)	C71—C72—H72B	109.5
C19—C20—Cl5	118.3 (4)	H72A—C72—H72B	109.5
C20—C21—C22	119.9 (5)	C71—C72—H72C	109.5
C20—C21—H21	120.0	H72A—C72—H72C	109.5
C22—C21—H21	120.0	H72B—C72—H72C	109.5
C23—C22—C21	120.2 (5)	O9—C73—C78	124.8 (4)
C23—C22—Cl6	120.2 (6)	O9—C73—C74	119.7 (4)
C21—C22—Cl6	119.7 (5)	C78—C73—C74	115.5 (4)
C22—C23—C24	121.1 (6)	C75—C74—C73	122.8 (5)
C22—C23—H23	119.4	C75—C74—Cl17	120.1 (4)
C24—C23—H23	119.4	C73—C74—Cl17	117.1 (4)
C23—C24—C19	120.4 (5)	C74—C75—C76	120.1 (5)
C23—C24—C25	118.4 (5)	C74—C75—H75	119.9
C19—C24—C25	121.2 (4)	C76—C75—H75	119.9
N3—C25—C24	127.2 (4)	C77—C76—C75	120.0 (5)
N3—C25—H25	116.4	C77—C76—Cl18	120.2 (5)
C24—C25—H25	116.4	C75—C76—Cl18	119.8 (4)
N3—C26—C27	111.5 (4)	C76—C77—C78	120.7 (5)
N3—C26—H26A	109.3	C76—C77—H77	119.7
C27—C26—H26A	109.3	C78—C77—H77	119.7
N3—C26—H26B	109.3	C77—C78—C73	120.8 (5)
C27—C26—H26B	109.3	C77—C78—C79	117.9 (5)
H26A—C26—H26B	108.0	C73—C78—C79	121.3 (5)
C26—C27—H27A	109.5	N9—C79—C78	127.5 (5)
C26—C27—H27B	109.5	N9—C79—H79	116.2
H27A—C27—H27B	109.5	C78—C79—H79	116.2
C26—C27—H27C	109.5	C81—C80—N9	106.7 (7)
H27A—C27—H27C	109.5	C81—C80—H80A	110.4
H27B—C27—H27C	109.5	N9—C80—H80A	110.4
O4—C28—C33	124.5 (4)	C81—C80—H80B	110.4
O4—C28—C29	119.9 (4)	N9—C80—H80B	110.4
C33—C28—C29	115.6 (4)	H80A—C80—H80B	108.6
C30—C29—C28	122.7 (4)	C80—C81—H81A	109.5
C30—C29—Cl7	119.1 (4)	C80—C81—H81B	109.5
C28—C29—Cl7	118.2 (4)	H81A—C81—H81B	109.5
C31—C30—C29	119.5 (5)	C80—C81—H81C	109.5
C31—C30—H30	120.3	H81A—C81—H81C	109.5
C29—C30—H30	120.3	H81B—C81—H81C	109.5
C32—C31—C30	120.3 (5)	O1—Co1—O3	173.95 (13)
C32—C31—Cl8	121.0 (4)	O1—Co1—O2	85.86 (12)
C30—C31—Cl8	118.7 (4)	O3—Co1—O2	89.02 (13)
C31—C32—C33	121.2 (5)	O1—Co1—N3	89.78 (14)
C31—C32—H32	119.4	O3—Co1—N3	93.10 (14)
C33—C32—H32	119.4	O2—Co1—N3	86.16 (14)
C32—C33—C28	120.6 (4)	O1—Co1—N1	91.12 (13)
C32—C33—C34	118.2 (4)	O3—Co1—N1	94.22 (14)
C28—C33—C34	121.2 (4)	O2—Co1—N1	174.70 (14)
N4—C34—C33	127.4 (4)	N3—Co1—N1	89.47 (15)
N4—C34—H34	116.3	O1—Co1—N2	90.16 (14)
C33—C34—H34	116.3	O3—Co1—N2	86.77 (14)
N4—C35—C36	111.1 (4)	O2—Co1—N2	91.81 (14)
N4—C35—H35A	109.4	N3—Co1—N2	177.96 (15)
C36—C35—H35A	109.4	N1—Co1—N2	92.56 (15)
N4—C35—H35B	109.4	O6—Co2—O4	173.70 (13)
C36—C35—H35B	109.4	O6—Co2—O5	85.31 (12)
H35A—C35—H35B	108.0	O4—Co2—O5	91.12 (12)
C35—C36—H36A	109.5	O6—Co2—N4	91.91 (14)
C35—C36—H36B	109.5	O4—Co2—N4	93.04 (14)
H36A—C36—H36B	109.5	O5—Co2—N4	86.50 (13)
C35—C36—H36C	109.5	O6—Co2—N5	90.22 (14)
H36A—C36—H36C	109.5	O4—Co2—N5	84.62 (13)
H36B—C36—H36C	109.5	O5—Co2—N5	90.65 (13)
O5—C37—C42	123.6 (4)	N4—Co2—N5	176.29 (14)
O5—C37—C38	121.1 (4)	O6—Co2—N6	91.49 (14)
C42—C37—C38	115.1 (4)	O4—Co2—N6	92.39 (13)
C39—C38—C37	122.9 (4)	O5—Co2—N6	175.19 (13)
C39—C38—Cl9	118.6 (4)	N4—Co2—N6	90.03 (15)
C37—C38—Cl9	118.5 (4)	N5—Co2—N6	92.95 (14)
C38—C39—C40	120.1 (4)	O8—Co3—O9	173.64 (14)
C38—C39—H39	120.0	O8—Co3—O7	84.50 (13)
C40—C39—H39	119.9	O9—Co3—O7	90.81 (14)
C39—C40—C41	120.3 (4)	O8—Co3—N7	91.60 (15)
C39—C40—Cl10	118.6 (4)	O9—Co3—N7	84.12 (15)
C41—C40—Cl10	121.0 (4)	O7—Co3—N7	90.45 (15)
C40—C41—C42	120.0 (5)	O8—Co3—N9	90.88 (17)
C40—C41—H41	120.0	O9—Co3—N9	93.39 (16)
C42—C41—H41	120.0	O7—Co3—N9	89.64 (17)
C41—C42—C37	121.5 (4)	N7—Co3—N9	177.51 (18)
C41—C42—C43	118.2 (4)	O8—Co3—N8	91.29 (15)
C37—C42—C43	120.2 (4)	O9—Co3—N8	93.41 (15)
N5—C43—C42	126.3 (4)	O7—Co3—N8	175.78 (15)
N5—C43—H43	116.9	N7—Co3—N8	89.92 (15)
C42—C43—H43	116.9	N9—Co3—N8	90.18 (18)
C45—C44—N5	117.9 (5)	C7—N1—C8	116.0 (4)
C45—C44—H44A	107.8	C7—N1—Co1	122.3 (3)
N5—C44—H44A	107.8	C8—N1—Co1	121.6 (3)
C45—C44—H44B	107.8	C16—N2—C17	115.7 (4)
N5—C44—H44B	107.8	C16—N2—Co1	120.4 (3)
H44A—C44—H44B	107.2	C17—N2—Co1	124.0 (3)
C44—C45—H45A	109.5	C25—N3—C26	117.1 (4)
C44—C45—H45B	109.5	C25—N3—Co1	122.6 (3)
H45A—C45—H45B	109.5	C26—N3—Co1	120.1 (3)
C44—C45—H45C	109.5	C34—N4—C35	116.9 (4)
H45A—C45—H45C	109.5	C34—N4—Co2	124.0 (3)
H45B—C45—H45C	109.5	C35—N4—Co2	119.1 (3)
O6—C46—C51	124.3 (4)	C43—N5—C44	118.5 (4)
O6—C46—C47	119.3 (4)	C43—N5—Co2	121.6 (3)
C51—C46—C47	116.3 (4)	C44—N5—Co2	119.9 (3)
C48—C47—C46	121.3 (5)	C52—N6—C53	115.3 (4)
C48—C47—Cl11	120.3 (4)	C52—N6—Co2	122.9 (3)
C46—C47—Cl11	118.4 (4)	C53—N6—Co2	121.8 (3)
C47—C48—C49	120.4 (5)	C61—N7—C62	117.8 (4)
C47—C48—H48	119.8	C61—N7—Co3	121.6 (3)
C49—C48—H48	119.8	C62—N7—Co3	120.6 (3)
C50—C49—C48	120.5 (5)	C70—N8—C71	118.7 (4)
C50—C49—Cl12	120.0 (4)	C70—N8—Co3	121.6 (3)
C48—C49—Cl12	119.5 (4)	C71—N8—Co3	119.6 (3)
C49—C50—C51	120.3 (5)	C79—N9—C80	115.4 (5)
C49—C50—H50	119.9	C79—N9—Co3	124.6 (4)
C51—C50—H50	119.9	C80—N9—Co3	119.9 (4)
C50—C51—C46	121.0 (4)	C1—O1—Co1	123.8 (3)
C50—C51—C52	118.8 (4)	C10—O2—Co1	119.9 (3)
C46—C51—C52	120.1 (4)	C19—O3—Co1	122.6 (3)
N6—C52—C51	127.1 (4)	C28—O4—Co2	126.4 (3)
N6—C52—H52	116.5	C37—O5—Co2	119.7 (3)
C51—C52—H52	116.5	C46—O6—Co2	125.1 (3)
C54—C53—N6	113.3 (5)	C55—O7—Co3	119.2 (3)
C54—C53—H53A	108.9	C64—O8—Co3	124.5 (3)
N6—C53—H53A	108.9	C73—O9—Co3	128.0 (3)

### Hydrogen-bond geometry (Å, º)

**Table:**

*D*—H···*A*	*D*—H	H···*A*	*D*···*A*	*D*—H···*A*
C44—H44*A*···Cl10^i^	0.97	2.80	3.748 (6)	168

Symmetry code: (i) −*x*+1, −*y*+1, −*z*.

## References

[bb1] Bruker (2004). *SMART*, *SAINT* and *SADABS* Bruker AXS Inc., Madison, Wisconsin, USA.

[bb2] Chen, F. Y., Zhang, S. H., Li, H. P., Zhang, L. J. & Zhang, Y. D. (2011). *Inorg. Chim. Acta*, **1006**, 142–146.

[bb3] Gupta, K. C. & Sutar, A. K. (2008). *Coord. Chem. Rev.* **252**, 1420–1450.

[bb4] Huang, Y. D., Zhang, S.-H., Qin, J. K. & Chen, F. L. (2010). *Acta Cryst.* E**66**, m1399. 10.1107/S1600536810040043 PMC3009188 21588832

[bb5] Park, J., Lang, K., Abboud, K. A. & Hong, S. (2008). *J. Am. Chem. Soc.* **130**, 16484–16485. 10.1021/ja807221s 19554722

[bb6] Sheldrick, G. M. (2008). *Acta Cryst.* A**64**, 112–122. 10.1107/S0108767307043930 18156677

[bb7] Sreenivasulu, B., Vetrichelvan, M., Zhao, F., Gao, S. & Vittal, J. J. (2005). *Eur. J. Inorg. Chem.* pp. 4635–4645.

[bb8] Zhang, S. H. & Feng, C. (2010). *J. Mol. Struct.* **977**, 62–66.

